# Genome-wide characterization and identification of Trihelix transcription factors and expression profiling in response to abiotic stresses in Chinese Willow (*Salix matsudana* Koidz)

**DOI:** 10.3389/fpls.2023.1125519

**Published:** 2023-03-03

**Authors:** Jie Yang, Zhixuan Tang, Wuyue Yang, Qianhui Huang, Yuqing Wang, Mengfan Huang, Hui Wei, Guoyuan Liu, Bolin Lian, Yanhong Chen, Jian Zhang

**Affiliations:** ^1^ School of Life Sciences, Nantong University, Nantong, China; ^2^ Key Laboratory of Landscape Plant Genetics and Breeding, Nantong, China

**Keywords:** genome-wide characterization, Trihelix family, Salix matsudana, submergence stress, RNA-Seq

## Abstract

Trihelix transcription factors (TTF) are a class of light-responsive proteins with a typical triple-helix structure (helix-loop-helix-loop-helix). Members of this gene family play an important role in plant growth and development, especially in various abiotic stress responses. *Salix matsudana* Koidz is an allotetraploid ornamental forest tree that is widely planted for its excellent resistance to stress, but no studies on its Trihelix gene family have been reported. In this study, the Trihelix gene family was analyzed at the genome-wide level in *S. matsudana*. A total of 78 *S. matsudana* Trihelix transcription factors (*SmTTFs*) were identified, distributed on 29 chromosomes, and classified into four subfamilies (GT-1, GT-2, SH4, SIP1) based on their structural features. The gene structures and conserved functional domains of these Trihelix genes are similar in the same subfamily and differ between subfamilies. The presence of multiple stress-responsive cis-elements on the promoter of the *S. matsudana* Trihelix gene suggests that the *S. matsudana* Trihelix gene may respond to abiotic stresses. Expression pattern analysis revealed that Trihelix genes have different functions during flooding stress, salt stress, drought stress and low temperature stress in *S. matsudana*. Given that *SmTTF30*, as a differentially expressed gene, has a faster response to flooding stress, we selected *SmTTF30* for functional studies. Overexpression of *SmTTF30* in *Arabidopsis thaliana* (Arabidopsis) enhances its tolerance to flooding stress. Under flooding stress, the leaf cell activity and peroxidase activity (POD) of the overexpression strain were significantly higher than the leaf cell activity and POD of the wild type, and the malondialdehyde (MDA) content was significantly lower than the MDA content of the wild type. Thus, these results suggest that *SmTTF30* enhances plant flooding tolerance and plays a positive regulatory role in plant flooding tolerance.

## Introduction

Trihelix transcription factors are a small family of transcription factors in plants, named because they contain three tandem helix structures (helix-loop-helix-loop-helix) in the DNA-binding structural domain. This domain specifically binds to the GT element, a light-response element on the DNA sequence, so the family is also known as the GT factor family (Green et al., 1987; Zhou, 1999). Generally, there are 1 or 2 domains present in the N-terminus or C-terminus of Trihelix proteins, which have highly consistent amino acid sequences and are strongly conserved among different member families. The Trihelix family is divided into five subfamilies in *Arabidopsis*: GT-1, GT-2, GTγ, SH4 and SIP1(Kaplan-Levy et al., 2012). The SH4 subfamily has a longer domain than the other subfamilies, but all other subfamily proteins have a 4^th^ alpha helix structure downstream of the conserved functional structural domain. In addition, the GT-2 subfamily contains a central α-helix domain, while the other subfamilies contain only a C-terminal α-helix domain (Kuhn et al., 1993; Ayadi et al., 2004; Xie et al., 2009). The conserved domain of the Trihelix transcription factor was found to overlap and be similar to the helix-turned-angle helix structure of the MYB transcription factor. Therefore, the domain of the Trihelix transcription factor contains the main features of the Myb transcription factor (Nagano, 2000; Song et al., 2021). The differences in structure may be related to the binding of target gene sequences, which also leads to functional differences in different subfamily genes of Trihelix.

Previous studies suggested that Trihelix proteins are plant-specific, but subsequent homologous sequence analysis revealed that Trihelix transcription factors are also present in the intestinal cavity of animals and insects (Dehesh et al., 1992). Currently, Trihelix transcription factor studies are reported mainly in plants. Compared with other gene families in plants, the Trihelix family is not large, with the number of members ranging from 30-60 in most species and approximately 100 in individual polyploid species. For example, there are 29 members of Trihelix in *Arabidopsis*, 41 in rice, 20 in chrysanthemums, 56 in black cottonwood, and 94 in hexaploid wheat (Jin et al., 2014; Song et al., 2016; Wang et al., 2016; Li et al., 2019; Xiao et al., 2019). Different members play important roles in plant growth and development, light regulation, and plant morphogenesis (Kaplan-Levy et al., 2012). Previous research has confirmed the role of Trihelix transcription factors in plant development. *Arabidopsis ASIL2* and rice *LOC-Os02g3610* genes are involved in regulating early embryo development. *AtASIL1* has a negative regulatory effect on seedling embryo shape and can maintain temporal control of seed germination(Willmann et al., 2011; Barr et al., 2012; Wan et al., 2015). Deletion of *Arabidopsis PTL* results in reduced petal number(Quon et al., 2017). Tomato *SlGT11* plays a role in the typing of floral organs and maintenance of floral characteristics (Yu et al., 2018). *Populus PtaGTL1* regulates stomatal development and plant water absorption (Yoo et al., 2019).

Trihelix family genes have received increasing attention from botanists for their important and critical role in abiotic stresses. The Trihelix family of transcription factors has been shown to play an important role in abiotic stresses such as salt stress, drought stress, cold stress and flooding stress (Kaplan-Levy et al., 2012). The *Arabidopsis* SIP1 subfamily member gene *AtAST1* produces physiological changes by regulating stress-responsive genes, ultimately improving salt and osmotic tolerance in transgenic plants (Xu et al., 2018). *CsGT-3b* in the cucumber and *BvM14-GT-3b* in the sugar beet both showed significant upregulation after salt stress (Wu et al., 2013; Wang et al., 2017). Two Trihelix family gene members of soybean, *GmGT-2A* and *GmGT-2B*, improved plant tolerance to salt, freezing and drought stresses (Xie et al., 2009). *ShCIGT*, a new member of the GT-1 subfamily, was identified in wild tomato, and overexpression lines showed better cold tolerance after cold stress. Phenotypic and physiological indicators suggest that *ShCIGT* improves tolerance by reducing cell membrane damage caused by cold stress (Yu et al., 2018). Seven members of the Trihelix transcription factor family were screened in maize as candidate genes for waterlogging tolerance and drought tolerance(Du et al., 2016). In addition, members of the Trihelix transcription factor SIP1 subfamily, *AtVFP3* and *AtVFP5*, were identified in *A. thaliana* to interact with Agrobacterium oncogenes to enhance plant tolerance to tumor growth (García-Cano et al., 2015).

*Salix matsudana* Koidz is an allotetraploid tree species of the genus Salix, which is widely planted in China because of its high resistance and adaptability to biotic and abiotic stresses and is known as the “Chinese willow” (Hou et al., 2019; Zhang et al., 2020; Liu et al., 2021). *S. matsudana* can be widely planted as a salt-tolerant species in areas with high soil water content, such as coastal mudflat wetlands, which also indicates that Willow has strong tolerance to survival in wetlands for a long time (Zhang et al., 2017). However, in its long life cycle, *S. matsudana* faces several challenges, including pests and diseases, salt stress, low temperature stress, drought stress, flooding and other stresses. Studies have been carried out to determine salt tolerance and Cd-resistence in *S. matsudana*, to identify salt tolerance genes (Yang et al., 2015; Yuan et al., 2019; Zhang et al., 2021; Chen et al., 2022a). However, there are currently few reports on the response of *S. matsudana to* flooding stress, the existence of submergence tolerant varieties, and genetic resources that could be used to improve flooding stress tolerance during the breeding program. Willows respond phenotypically and physiologically to flooding, yet the molecular mechanisms underlying their response remain unknown. In this study, we systematically analyzed the molecular evolution, gene structure, cis-elements, conserved motifs and expression patterns of Trihelix gene family members in *S. matsudana*. Then, we proximally characterized the function of *SmTTF30* under flooding stress. These results indicate that *SmTTF30* enhances plant submergence tolerance, which helps us to elucidate the response and regulatory mechanism of *S. matsudana* to submergence stress and provide a theoretical basis for molecular breeding.

## Materials and methods

### Plant material, growth conditions and treatment

The plant materials in this study included the arbor willow ‘Suliu 795’ (Salix × jiangsuensis ‘J795’) and two genotypes of *Arabidopsis*, Col-0 (WT) and *SmTTF30*/WT (OE). All *Arabidopsis* lines were germinated on half-strength Murashige and Skoog media supplied with 2% sucrose (1/2 MS) for 10-14 days, and then the seedlings were planted on mixed soil (50% peat soil and 50% vermiculite). One-year-old stem cuttings (length, 8-10 cm; coarse, 2-3 cm) of ‘suliu 795’ were immersed in water, which was collected from the botany garden of Nantong University (Nantong, China). All materials were grown in an artificial climate chamber (16 h day/8 h night, 22°C day/18°C night). Five-week-old *Arabidopsis* plants were subjected to flooding treatment with the water surface 3 cm above the plants. After treatment for 0, 4 h, 12 h, 24 h, 48 h, 3 days, 5 days, recovery 4 h and 24 h, and leaves were collected. Two-week-old willows were incubated with 20% PEG6000, 200 mM mannitol solutions and low temperature (4°C). After treatment for 0, 4 h, 12 h, 24 h, and 48 h, adventitious roots were collected. This experiment was independently repeated three times. All samples were subsequently frozen and stored at -80°C for RNA extraction. Flooding stress transcriptome data are under 4 h, 12 h, 24 h and 48 h of flooding treatment, and salt stress transcriptome data are after 0 h, 4 h, 8 h and 12 h of 200 mM NaCl treatment.

### Identification and characterization of *SmTTF*

Sequences of *Arabidopsis thaliana*, *Oryza sativa*, *Populus trichocarpa*, and *Salix purpurea* were downloaded from JGI (https://www.phytozome.net/). *S. matsudana* sequences were obtained from our sequencing, and assembly results were obtained by Roche/454 and Illumina/HiSeq-2000 sequencing technologies(Zhang et al., 2020). *Arabidopsis* trihelix family member sequences were downloaded from the Plant Transcription Factor Database PlantTFDB (http://planttfdb.gao-lab.org/) (Zhang et al., 2011). The *P. trichocarpa* Trihelix gene sequence was obtained from the article(Wang et al., 2016). Based on two BLASTp methods, the 34 triheix protein sequences of *Arabidopsis* retrieved from the Plant Transcription Factor Database (PlantTFDB) were used as queries to obtain the possible Trihelix proteins in the *S. matsudana* genome by BLASTp search with a cutoff E-value of 1.0×10^-10^. All the trihelix candidate proteins were checked using the Conserved Domain Database (CDD) of NCBI and SMART database (Lu et al., 2020). ExPASy was used to determine the basic physical and chemical characteristics of 78 trihelix proteins, such as the molecular weight (MW), isoelectric point (pI) and amino acid sequence length (Wilkins et al., 1999). Cell-PLoc was used to predict subcellular localization (Chou and Shen, 2008). DEGs were obtained from the results of transcriptome data. DEGs were identified using the DEGseq R package with filtering criteria of false discovery rate (FDR)<0.01 and fold-change ≥2. Then we entered the gene names of TTFs to search among all DEGs.

### Phylogenetic analysis and classification of *SmTTF* genes

*Arabidopsis* and *Populus* Trihelix genes were extracted from their genomes according to previous reports. The amino acid sequences of the Trihelix gene from three plants were used to construct a neighbor-joining (NJ) phylogenetic tree by MEGA X software with default parameters (Kumar et al., 2018). The bootstrap value was set to 1,000. The tree was embellished by web-based software ChiPlot(https://www.chiplot.online/tvbot.html).

### Gene structure, conserved motif structure and promoter cis-element analysis

The intron-exon structures of *SmTTFs* were obtained based on the gene annotation Gff3 files we assembled. To characterize the structure of *SmTTF* proteins, the conserved motifs were analyzed using the online tool MEME with the following parameters: motif 10 and width between 6-50 amino acid residues (Bailey and Elkan, 1994). Cis-elements on the promoter (-2,000 bp upstream ATG) were predicted on the PlantCARE website. Graphics of structures were drawn using TBtools software (Chen et al., 2020).

### Gene position on chromosomes and collinearity analysis

Location and chromosome length information of every *SmTTF* gene was obtained from the gene annotation Gff3 file. TBools software was used to draw the chromosomal distribution image of *SmTTF* genes. The detection and identification of gene duplication events in *SmTTF* genes were performed using multiple collinear scanning toolkits (MCScanX) with default parameters. MCScanX was also used with default settings to analyze the syntenic relationship of the trihelix family genes among the *S. matsudana*, *A. thaliana*, *O. sativa*, *P. trichocarpa*, and *S. purpurea* genomes. After obtaining duplicated gene pairs, the synonymous substitution rate (K_s_) and nonsynonymous substitution rate (K_a_) of gene pairs were calculated using the ‘Simple Ka/Ks Calculator’ of TBtools.

### RNA extraction and real-time quantitative PCR

RNA from the plant tissue of all stress-treated materials was extracted using the RNAprep Pure Plant Plus Kit (TIANGEN, DP441, Beijing, China). The RNA was used to synthesize first-strand cDNA with the PrimeScript RT Reagent Kit (Takara, RR037Q, Beijing, China). The primers used for qRT-PCR are listed in **Supplementary Table 2**. The program was performed using ABI7500 according to the manufacturer’s instructions. PCR mixes were made following the protocols of the UltraSYBR Mixture(CWBIO, CW2601S, Taizhou, China). The expression levels were calculated using 2^-ΔΔCt^ and compared to the internal control and CK sample (Livak and Schmittgen, 2001).

### Cloning of *SmTTF30*, vector construction and transformation of *A. thaliana*

Leaf RNA of *S. matsudana* and first-strand cDNA were obtained using the methods described above. *SmTTF30* was amplified by 2×Es Taq MasterMix (CWBIO, CW0690L, Taizhou, China) using the primers listed in **Supplementary Table 2**. The PCR products were cloned into a PMD18-T vector (Takara, 6011, Beijing, China) according to the manufacturer’s protocols. pWM101-*35S:SmTTF30* was constructed using an infusion strategy (ClonExpress II One Step Cloning Kit, C112-01, Vazyme, Nanjing, China). The pWM101-*35S:SmTTF30* construct was first transformed into *Arabidopsis* WT (Col-0) through the agrobacterium-mediated (GV3101) floral dip method reported previously (Clough and Bent, 1998). The positive *SmTTF30*/WT(OE) T1 plants were screened using half strength (1/2) MS with 20 mg/L hygromycin and genomic PCR with *SmTTF30*-specific primers (**Supplementary Table 2**).

### Evans blue stain and detection of physiological paramenters

Leaves of WT and three independent *SmTTF30* lines were immersed in 0.25% Evans Blue solution, rinsed with water and placed in anhydrous ethanol for decolorization (Jacyn Baker and Mock, 1994). The leaves were laid flat and scanned with a scanner. All leaves were homogenized using liquid nitrogen. The activity of peroxidase (POD, A084-3-1, NJJCBIO, Nanjing, China) and the content of malondialdehyde (MDA, A003-3-1, NJJCBIO, Nanjing, China) were detected using the respective kits according to the manufacturer’s instructions. All treatments were applied to at least three replicates.

### Data analysis

Origin 2018 (Originlab, MA, USA) was used to construct graphs of the data. SPSS version 25 statistical software (SPSS, NY, USA) was used to detect statistically significant differences.

## Results

### Identification, phylogenetic analysis and classification of *SmTTFs*

In this study, 78 Trihelix family members were identified in the *S. matsudana* genome with reference to known Trihelix protein sequences in *Arabidopsis*, and every gene was confirmed to contain the Trihelix structural domain. The 78 Trihelix genes were named *SmTTF01-SmTTF78* according to their position on the chromosome. Among the 78 members identified, the *SmTTF* gene encoded proteins with lengths ranging from 206 aa to 769 aa, molecular weights ranging from 24.48 kDa to 83.64 kDa, and isoelectric points ranging from 4.55 to 10.1, with the smallest being *SmTTF34* and the largest being *SmTTF70*. The subcellular localization prediction results showed that all proteins could localize to the nucleus, and five proteins localized to chloroplasts, *SmTTF05*, *SmTTF39*, *SmTTF40*, *SmTTF55 and SmTTF78*, which may be related to photosynthesis. *SmTTF40* and *SmTTF68* were also localized to the peroxisome, and we speculated that those proteins are related to photosynthesis, metabolism and developmental processes. **Supplementary Table 1** listed basic information about all Trihelix members in *S. matsudana*.

**Figure 1:**
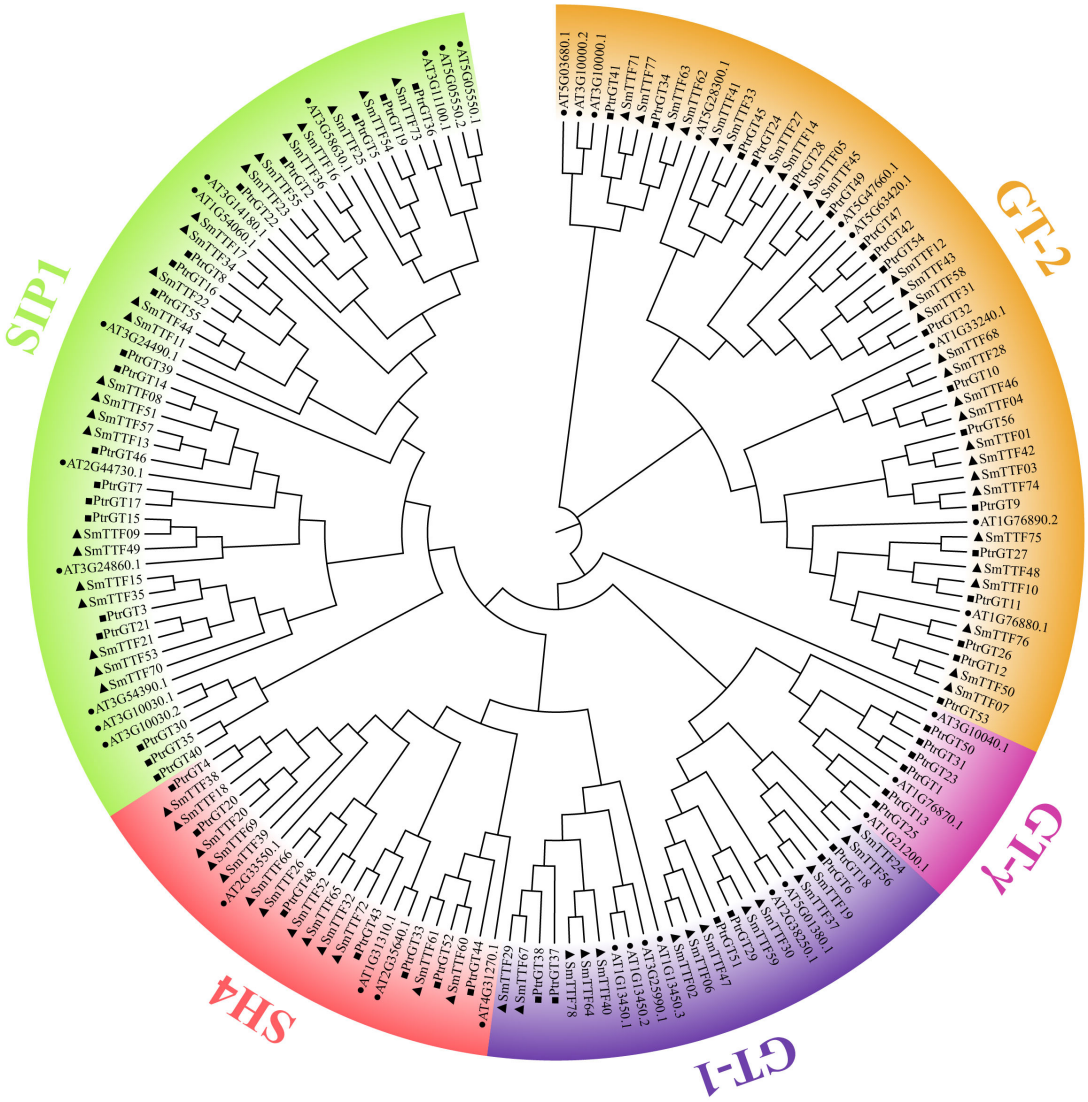
Phylogenetic tree of trihelix proteins in *S. matsudana, A. thaliana and P. trichocarpa.* Different colored branches represent different subfamilies. *S. matsudana*, *A. thaliana and P. trichocarpa* are marked as triangles, circles and squares, respectively.

To investigate the phylogenetic relationships among the Trihelix proteins in *Arabidopsis*, poplar and salix, a neighbor-joining phylogenetic tree was constructed using 168 Tihelix sequences, including 34, 56 and 78 sequences from *Arabidopsis*, poplar and salix, respectively (**Figure 1**). Among these species, GT-2, with 28 *SmTTF* family members, was the largest subfamily, whereas SH4 was the smallest clade, with 13 *SmTTF* family members. The SIP1 and GT-1 clusters contained 23 and 14 *SmTTF* members, respectively. There was no GTγ family member in *S. matsudana*; therefore, we hypothesized that *S. matsudana* is conservative in evolution, which also indicated evolutionary differences between *S. matsudana* and other species. The phylogenetic tree showed that the evolutionary relationship between GTγ, GT1 and GT2 was relatively close and intermediate, indicating that GTγ evolved from GT1 and GT2, and the functions of the three subfamily genes were similar.

### Gene structure and conserved motifs in *S. matsudana*

**Figure 2:**
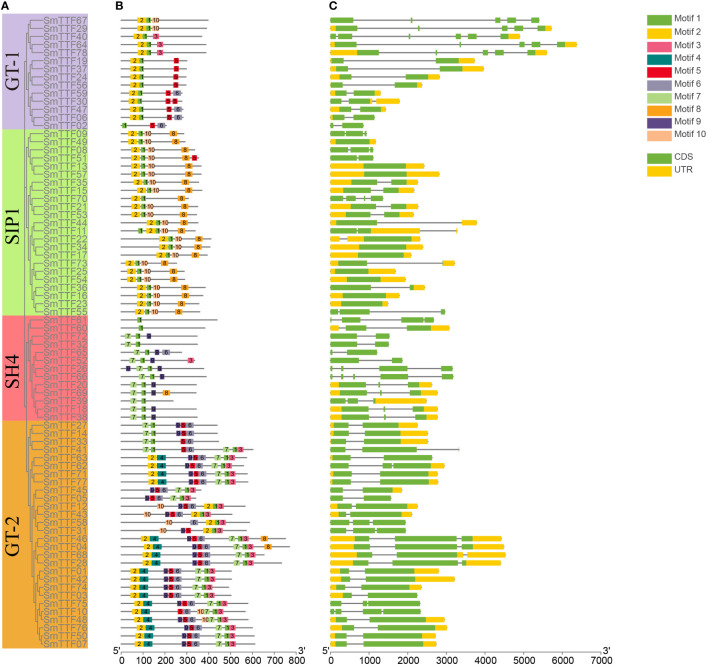
Structures and conserved motifs of SmTTF genes. **(A)** The phylogenetic tree was constructed based on the amino acid sequences of *S. matsudana* trihelix proteins. Different colored branches represent different subfamilies. **(B)** The motif compositions of *S. matsudana* Trihelix proteins. Motifs 1-10 are marked in different colored boxes. **(C)** Gene structure of the *S. matsudana* trihelix family. CDS, introns, and untranslated regions are marked by green boxes, gray lines, and yellow boxes, respectively.

The conserved motifs of the Trihelix gene of *S*. *matsudana* were analyzed by the MEME Suite tool, and a total of 10 conserved motifs were identified. The visualization results were shown in **Figures 2A, B**, where the conserved structural domains of the same subfamilies were similarly distributed. All genes contained Motif1, which was annotated as a Myb/SANT-like DNA-binding domain because of its overlap and similarity to the helix-turned-angle helix structure of the MYB transcription factor. GT-1 and SIP1 subfamily genes all contained Motif1 and Motif2, located at the 5’ end. The GT-1 subfamily of genes also included motifs 3, 5, 6, and 10. In the SIP1 subfamily, motifs 1, 2 and 10 were located at the 5’ end, and motif 8 is located at the 3’ end. The SH4 subfamily genes also contained motifs 3, 6, 7, and 9, with motif 7 located at the 5’ end and motif 9 at an intermediate position. The GT-2 subfamily genes also included motifs 2, 3, 4, 5, 6, 7, and 9, with motifs 2 and 4 concentrated at the 5’ end, motifs 3 and 7 concentrated at the 3’ end, and motifs 5, 6, and 9 concentrated at the intermediate position.

The structure of these Trihelix genes was further analyzed to characterize the *S. matsudana* Trihelix gene family (**Figure 2C**). The number of exons of the Trihelix gene in *S. matsudana* was distributed between 1 and 6, with the highest average number of exons in the GT-1 and GT-2 subfamilies and the lowest in the SIP1 subfamily. We found that the SIP1 gene had only 1-2 exons, 5 of the 13 genes in the SH4 subfamily had 3 exons, and 3 genes had more than 3 exons. Nine and 19 genes in the GT-1 and GT-2 subfamilies, respectively, contained two exons, five of the GT-1 genes had 5-6 exons, seven of the GT-2 genes contained three exons, and one had four more exons. Members of the same subfamily were observed to share a similar genetic structure.

### Chromosome distribution, evolutionary and collinearity analysis of the *SmTTFs*

**Figure 3:**
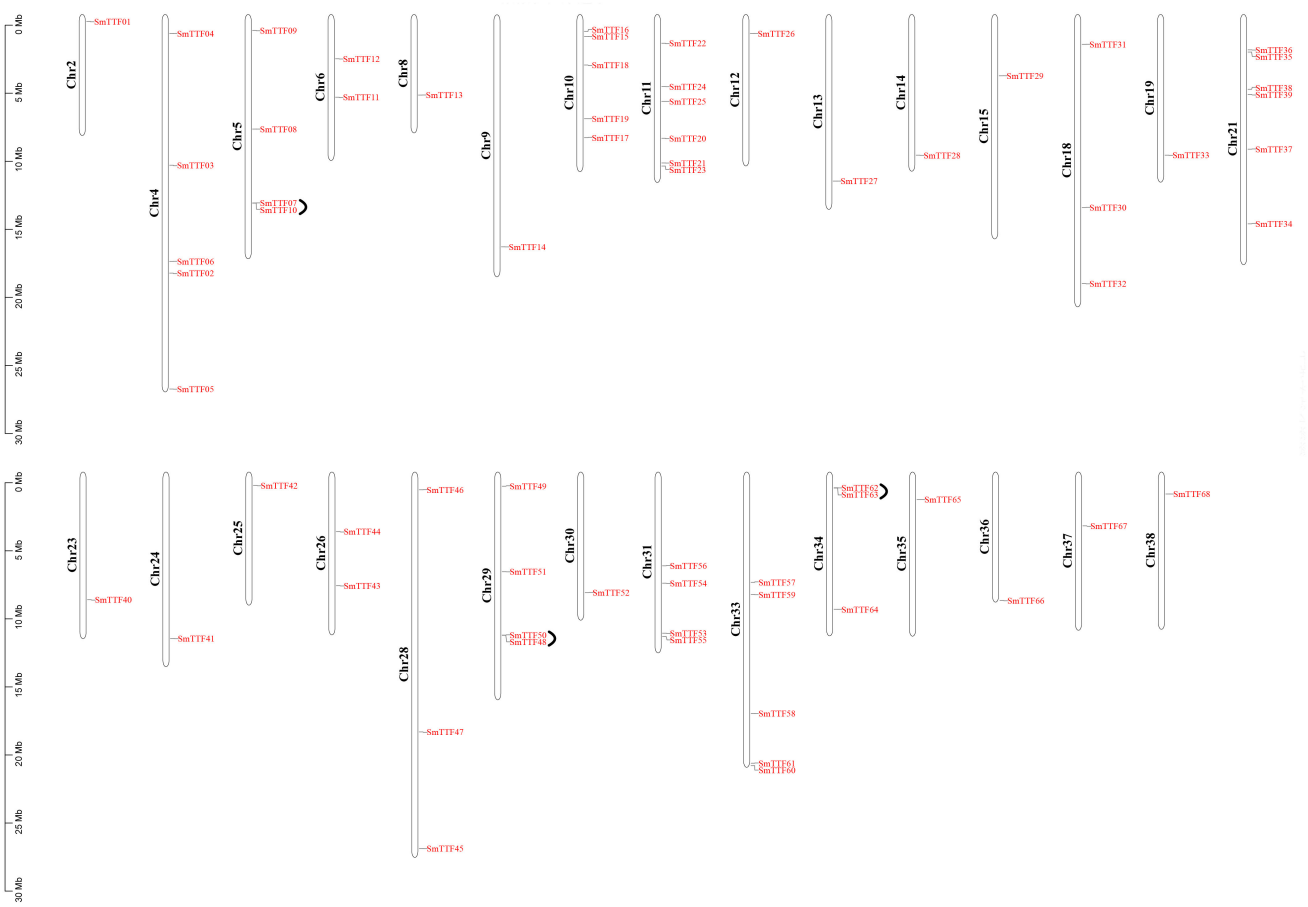
Chromosomal locations and tandem duplication of *S. matsudana* trihelix genes. The black lines indicate tandem duplicated trihelix gene pairs. The chromosome number is indicated to the left of each chromosome.

**Figure 4:**
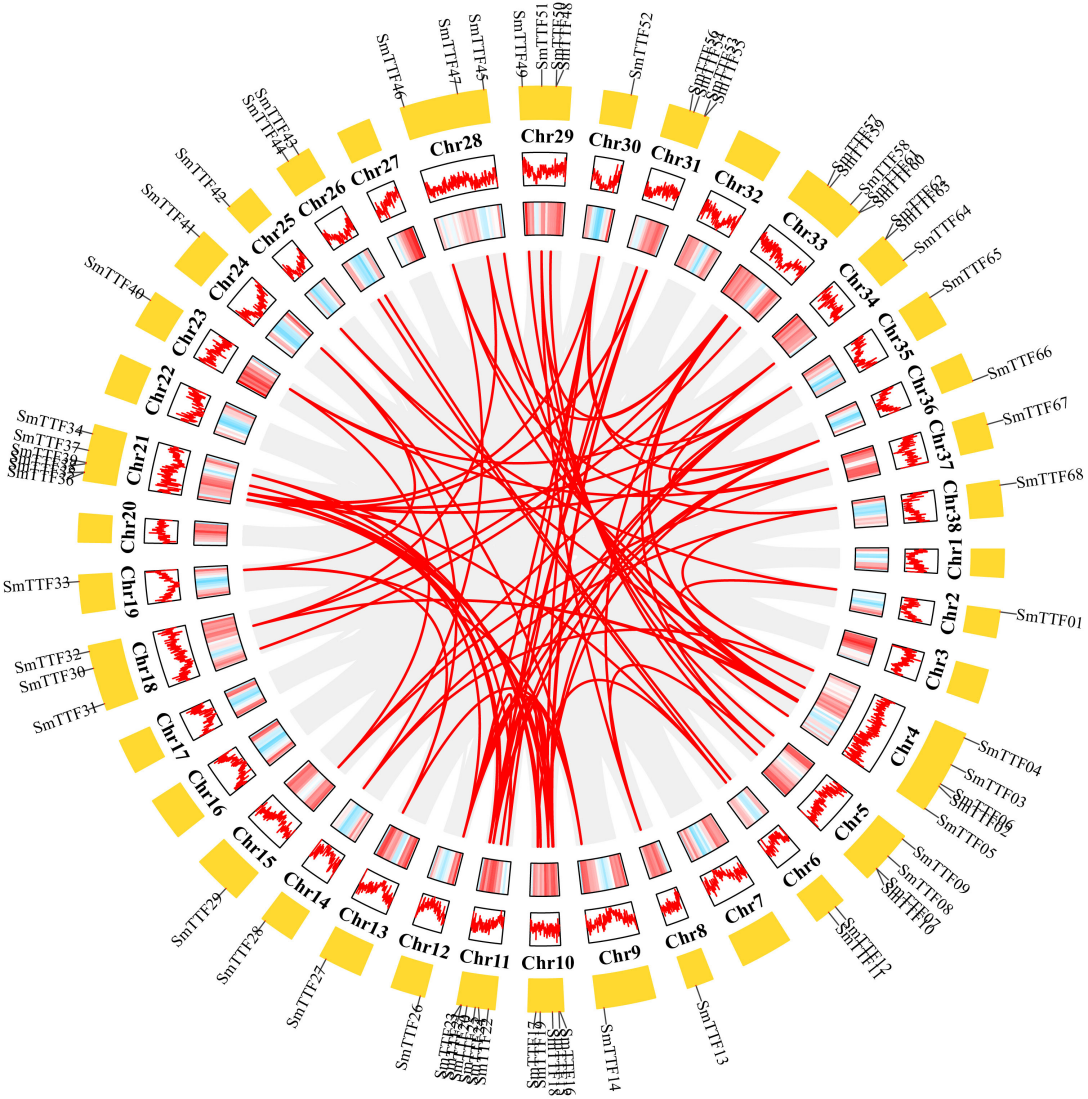
Schematic representations of the segmental duplication and interchromosomal relationships of SmTTF genes. Gray lines indicate all syntenic gene pairs in the *S. matsudana* genome, and red lines indicate syntenic relationships between *SmTTF* genes. Gene density across chromosomes is indicated by a hot map (inner circle) and column map (medium circles), and the outer circle shows the length of chromosomes.

Chromosomal localization of the identified Trihelix genes was performed (**Figure 3**), and 68 of the 78 *SmTTF* genes were distributed on 29 of the 38 chromosomes of *S. matsudana*. Chromosomes 11 and 21 contained the highest number of genes with six genes, and chromosomes 2, 8, 9, 12, 13, 14, 15, 19, 23, 24, 25, 30, 35, 36, 37, and 38 all had only one gene distribution. Trihelix genes from different subfamilies were mostly randomly distributed; for example, 11 of the 13 SH4 subfamily genes were located on Chr10, Chr11, Chr12, Chr18, Chr21, Chr30, Chr33, Chr35, and Chr36. The analysis identified 111 pairs of duplicated genes, including three pairs of tandem repeats, *SmTTF07* and *SmTTF10*, *SmTTF48* and *SmTTF50*, and *SmTTF62* and *SmTTF63* tandem repeats. Intragenomic covariance analysis identified the remaining 108 pairs as segmental repeats (**Figure 4**). The Ka/Ks of all duplicated gene pairs were less than 1 (**Supplementary Table 3**), indicating that these genes may have experienced strong purifying selection pressure during evolution.

**Figure 5:**
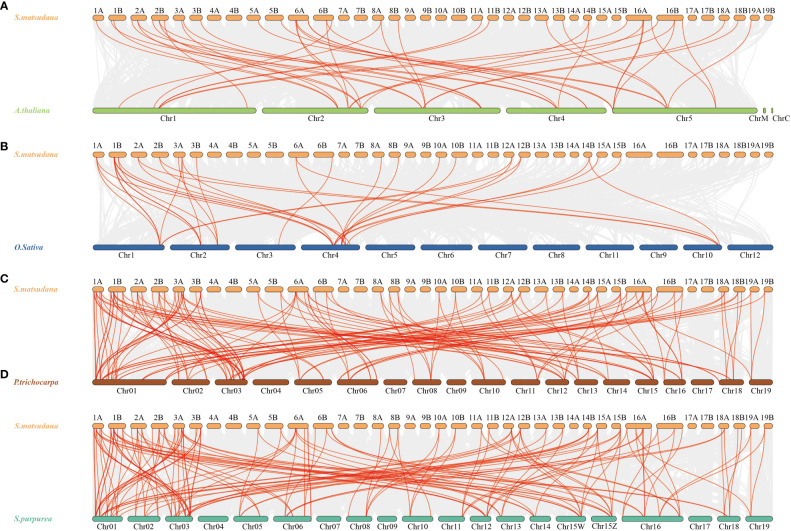
Synteny analysis of SmTTF genes between *S. matsudana* and four related species, *A*. *thaliana*, *O. sativa*, *P. trichocarpa* and *S. purpurea*. **(A)** Synteny analysis of SmTTF genes between *S. matsudana* and *A*. *thaliana*. **(B)** Synteny analysis of SmTTF genes between *S. matsudana* and *O. sativa*. **(C)** Synteny analysis of SmTTF genes between *S. matsudana* and *P. trichocarpa*, **(D)** Synteny analysis of SmTTF genes between *S. matsudana* and *S. purpurea.* Gray lines in the background indicate the collinear blocks within *S. matsudana* and other plant genomes, whereas red lines highlight syntenic SmTTS gene pairs.

To gain a closer understanding of the replication mechanism of TriHelix gene family members in *S. matsudana*, we constructed covariance maps of *S. matsudana* and *A. thaliana*, *O. sativa*, *P. trichocarpa* and *S. purpurea* (**Figure 5**). The results of covariance analysis showed that 37 *SmTTF* genes in *S. matsudana* showed covariance with 16 genes in *Arabidopsis* (**Figure 5A**). Twenty *SmTTF* genes showed covariance with 9 genes in rice (**Figure 5B**). Sixty-four SmTTF genes showed collinearity with 40 genes in poplar and 41 genes in *S. purpurea* with 65 SmTTF genes (**Figures 5C, D**). The homologous gene pairs of *S. matsudana* with *A. thaliana*, *O. sativa*, *P. trichocarpa* and *S. purpurea* were 45, 35, 142 and 147, respectively, indicating a high species affinity between *S. matsudana* and *P. trichocarpa* and *S. purpurea*.

### Cis-elements on the promoter of *SmTTFs*

**Figure 6:**
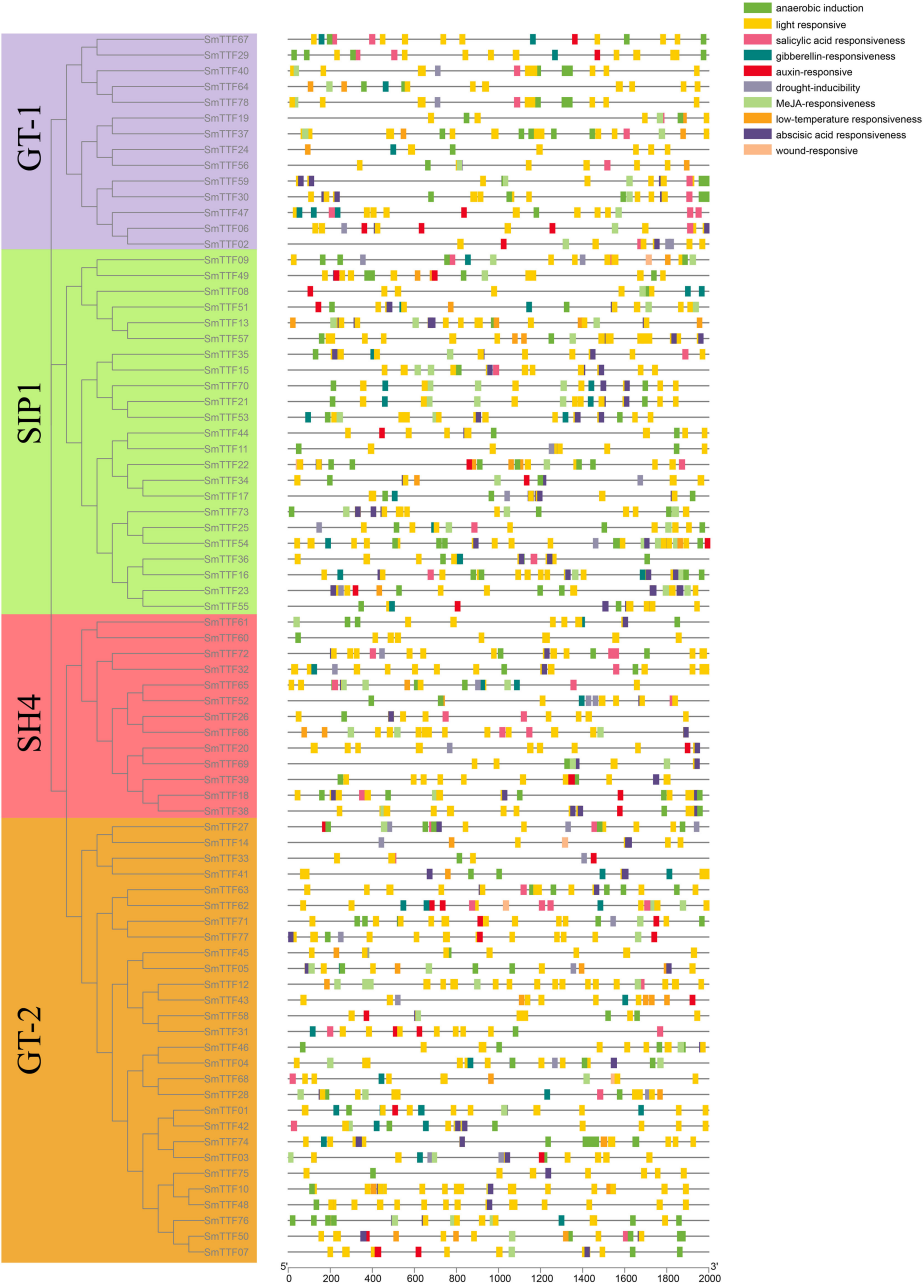
Distribution of predicted cis-acting elements in the promoter region. Elements are indicated as rectangles, and other sequences are indicated as lines.

To more closely analyze the functions of the Trihelix gene in *S. matsudana* that have diverged during evolution, especially the plant response to abiotic stresses. We submitted a 2000 bp sequence upstream of each *SmTTF* translation start site to the PlantCARE database to search for specific cis-acting elements. All Trihelix genes in *S. matsudana* contained at least 10 cis-acting elements related to the light response, which was also consistent with the light response properties of the Trihelix transcription factor family (**Figure 6**)(Kaplan-Levy et al., 2012). We also identified cis-elements associated with abiotic stress. The hypothermia response element, drought response element, and hypoxia response element were present in 30, 27, and 69 gene promoter regions, respectively. Hormone-related regulatory elements were also identified, including those related to auxin, gibberellin, abscisic acid and salicylic acid, of which 77 genes contained at least one hormone-related gene.

### Different *SmTTFs* respond to abiotic stresses differently

**Figure 7:**
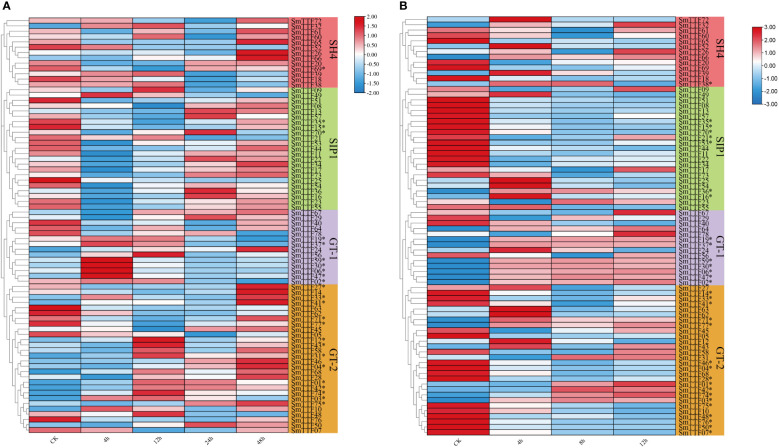
Heatmap of the expression profiles of *Salix matsudana* trihelix genes under submergence stress and salt stress. **(A)** Expression profiles of trihelix genes under flooding stress. **(B)** Expression profiles of trihelix genes under salinity stress. Differentially expressed genes are marked with *. The expression values (Fragments per kilobase for a million reads, FPKM) for each gene were log2 transformed before generating the heatmap.

To investigate the role of Trihelix genes in abiotic stress, we analyzed the expression pattern of *SmTTFs* from submergence stress and salt stress transcriptome data. A total of 25 and 32 *SmTTF* genes were identified as differentially expressed genes under flooding stress and salt stress, respectively, using fold change ≥ 2 and FDR < 0.01 as screening criteria. FPKM values of these genes were entered using TBtools software to construct heatmaps showing gene expression patterns under flooding stress (**Figures 7A, B**). The A-plot showed the expression pattern of 78 *SmTTF* genes under flooding stress, with 25 differentially expressed genes (* labeled). Fourteen genes in the GT-2 subfamily were differentially expressed genes, seven differentially expressed genes in the GT-1 subfamily, and three and one in the SIP1 and SH4 subfamilies, respectively. The GT-1 subfamily genes *SmTTF02*, *06*, *19*, *30*, *37*, *47*, and *59* had the highest gene expression levels after 4 h of submergence. Five of the GT-2 subfamily genes were downregulated after flooding (*SmTTF03*, *05*, *48*, *62*, *63*), and differentially expressed genes in the SIP1 and SH4 subfamilies were also downregulated after flooding and upregulated again at 48 h of flooding.

Panel B showed the expression pattern of *SmTTF* genes under salt stress, with 17 differentially expressed genes in the GT-2 subfamily, seven DEGs in both the GT-1 and SIP1 subfamilies, and only one DEG in the SH4 subfamily. The differentially expressed gene *SmTTF38* in the SH4 subfamily had the highest expression at 4 h of stress, followed by a gradual decrease in expression. The differentially expressed genes *SmTTF15*, *21*, *35*, *53*, and *70* in the SIP1 subfamily had downregulated gene expression after salt stress, whereas *SmTTF16* and *36* had upregulated expression at 4 h and subsequently downregulated expression. The differentially expressed genes in the GT-1 subfamily were upregulated after salt stress and remained relatively stable from 4-12 h. *SmTTF01*, *03*, *31*, *42* and *74* in the GT-2 subfamily were upregulated in the gene table at 8 h of stress, whereas the remaining differentially expressed genes were, in contrast, downregulated in expression after salt stress. The expression changes in these genes at different times may had direct or indirect regulatory relationships with other genes. Transcriptomes suggested that most Trihelix family genes play different roles at specific times during the response of willow to submergence stress and salt stress.

**Figure 8:**
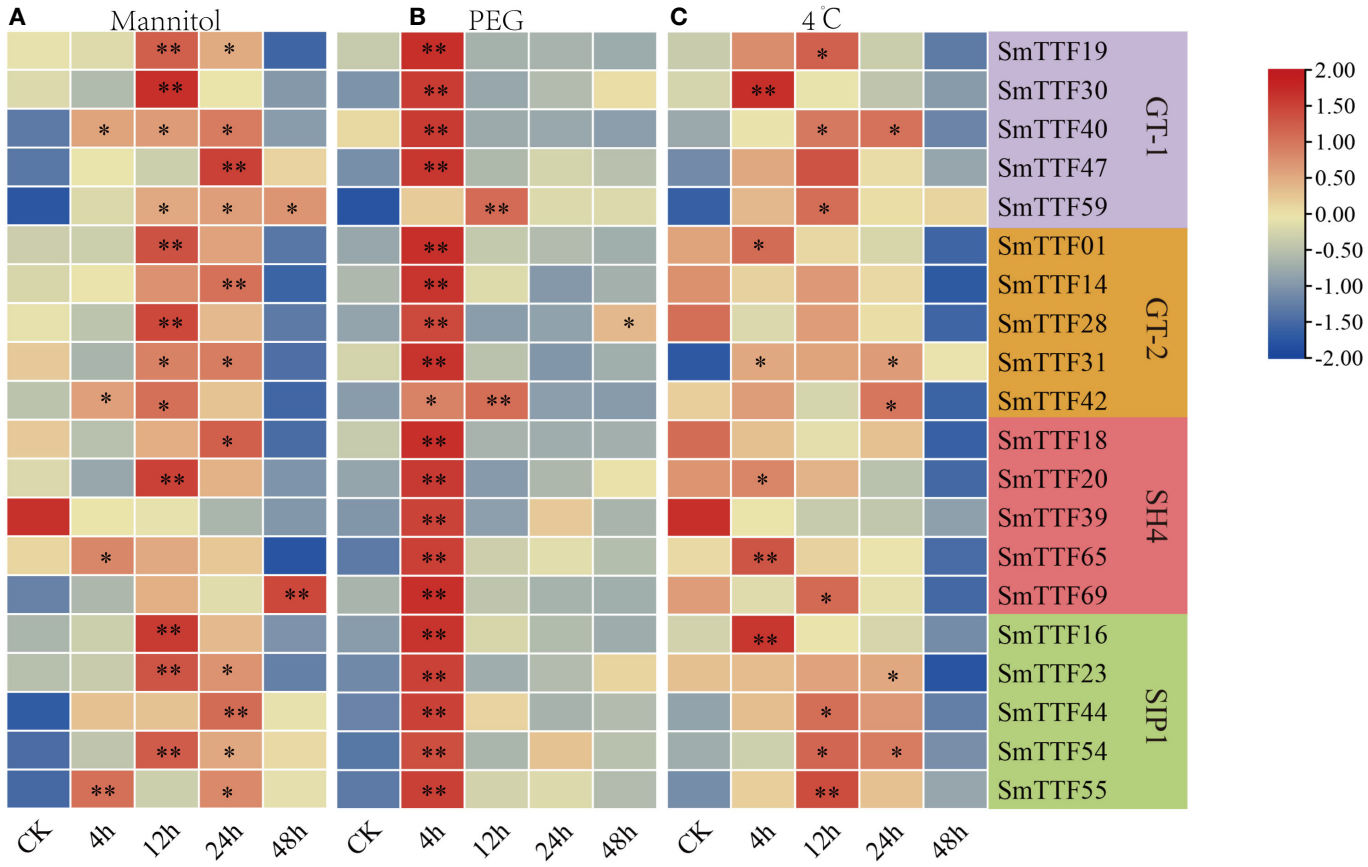
The expression profiles of 20 selected trihelix genes in *S. matsudana* under other abiotic stresses by qRT-PCR. **(A)** The expression profiles under 200 mM mannitol solutions. **(B)** The expression profiles under 20% PEG6000. **(C)** The expression profiles under 4°C. All roots from plants were treated for 4 h, 12 h, 24 h and 48 h. The fold change in expression level is depicted by a heatmap. Data are average values ± SD (*n* = 3) calculated from three independent experiments. Asterisks indicate significant differences from WT (*p < 0.05, **p < 0.01 by Student’s *t*-test).

We treated 3-week-old cuttings of Suliu 795 with 200 mM mannitol, 20% PEG6000 and 4°C low temperature stress and selected 20 genes of different subfamilies for analysis (**Figure 8**). This result indicated that the expression levels of response members differ under different abiotic stresses. *SmTTF* genes showed upregulated expression at 4-24 h under mannitol and low temperature treatment, while *SmTTF59* of the GT-1 subfamily and *SmTTF69* of the SH4 subfamily showed the highest expression at 48 h of treatment under mannitol treatment. The expression of *SmTTF14* and *28* of the GT-2 subfamily and *SmTTF18*, *20* and *39* of the SH4 subfamily were gradually reduced under low temperature treatment. Under PEG treatment, all of the *SmTTF* genes, except *SmTTF59* and *SmTTF42*, were determined to show the highest expression at 4 h of submergence, followed by a gradual decrease in expression. The results showed that *SmTTF* genes can respond to submergence stress, salt stress, drought stress and low temperature stress, and the potential functions of this gene family in responding to and regulating abiotic stresses were valuable for further study.

### *SmTTF30* enhances flooding tolerance in *A. thaliana*

**Figure 9:**
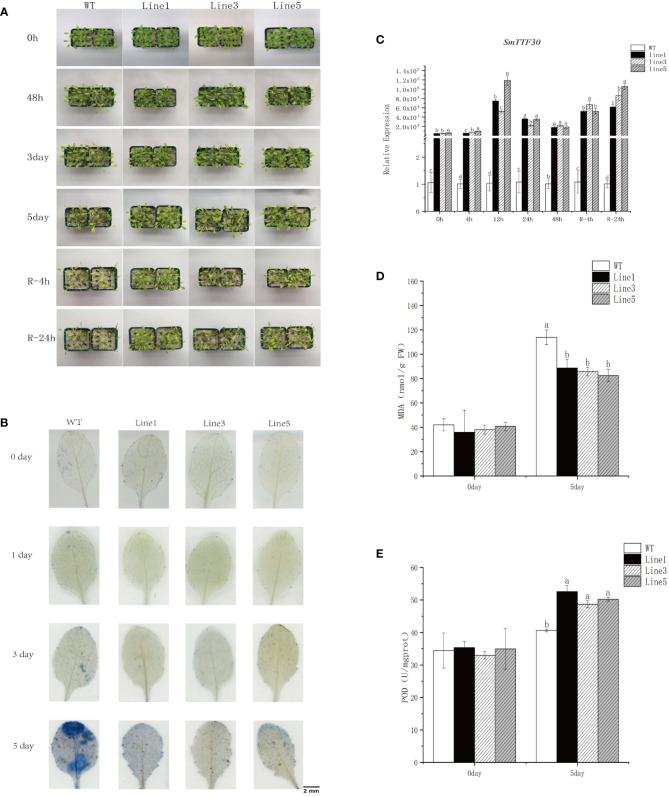
Submergence tolerance assay of *SmTTF30* overexpression lines (Line1, Line3, Line5) and wild type (WT). **(A)** Five-week-old plants were subjected to submergence stress for 6 days. **(B)** Evans blue staining. **(C)** qRT-PCR analysis of transgenic and wild-type plants. **(D)** MDA content. **(E)** POD activity. The mean value and standard deviation were obtained from three independent experiments. The data represent the mean ± SD of three biological repeats with three measurements per sample. Data were analyzed by one-way analysis of variance followed by Duncan’s test. Different letters represent statistically significant differences (*p* < 0.05).

Transcriptome and qRT-PCR data showed that *SmTTF30* responded significantly to a variety of abiotic stresses. Our previous study showed that *SmTTF30* of the *S. matsudana* Trihelix gene family was identified as a flooding tolerance hub gene, and analysis of transcriptomic data revealed that the gene was significantly induced at 4 h of flooding (Chen et al., 2022b). Evolutionary tree analysis revealed that this gene is closely related to *Arabidopsis HRA1* (At3g10040), and the study demonstrated that HRA1 can be involved in *Arabidopsis* in response to flooding stress(Giuntoli et al., 2014). To investigate whether *SmTTF* was involved in flooding stress, we constructed the structure of 35S:*SmTTF30* and transformed it into *Arabidopsis* WT (Col-0). Overexpression lines were successfully obtained by genotypic identification and expression analysis of the transformed lines. Five-week-old plants of wild-type and three transgenic lines were subjected to 5 days of submergence stress. After flooding stress, the gene expression of the overexpression lines was significantly upregulated, and the gene expression pattern showed a gradual upregulation after flooding, reaching the highest level at 12 h, followed by a gradual downregulation and then a significant upregulation after reoxygenation (**Figure 9C**). Under normal conditions, there was no difference in growth between the transgenic lines and the wild-type plants. On Day 5 of submergence, wild-type *Arabidopsis* leaves showed significant yellowing and hyalinization with curled leaf edges, whereas the overexpression lines grew well (**Figure 9A**). Immediately after reoxygenation, the overexpression lines grew in a better state than the wild type after 4 h and 24 h of reoxygenation. The results of Evans blue staining showed that at 5 days of flooding, the blue area of wild-type *Arabidopsis* leaves was larger than the blue area of the overexpression lines, and the leaf cell activity was reduced and cell death occurred, while the overexpression lines had less cell damage in the leaves (**Figure 9B**). In addition, the malondialdehyde content increased after flooding stress, and the MDA content was significantly higher in the WT than in the OE lines (**Figure 9D**). The POD content also increased after flooding and was significantly higher in the OE than in the WT lines (**Figure 9E**). In addition, we determined the expression levels of hypoxia-responsive genes. The expression levels of ADH1, PCO2, SUS1, and SUS4 in the overexpression strain were significantly higher than those of the wild type after 5 days of inundation (**Figure S1**). Taken together, these results suggested that *SmTTF30* overexpression in *Arabidopsis* can improve plant tolerance to flooding stress.

## Discussion

An increasing number of studies have demonstrated that Trihelix genes play an important role in different growth and developmental processes such as flowering, stomatal, epidermal hair, embryo and seed development in plants, as well as in abiotic stress responses such as disease, salt stress, drought stress and cold stress (Kaplan-Levy et al., 2012). In this study, we obtained 78 members of the Trihelix gene family by using two BLASTp methods, using the members of the Trihelix gene family as a reference. This number is greater than the number of Trihelix genes reported in the literature for *A. thaliana*, *O. sativa* and *P*. *trichocarpa* (34, 31, 56), which echoes *S. matsudana* as an allotetraploid species. We identified a total of 111 pairs of duplicated genes, of which 3 pairs (*SmTTF07/10*, *SmTTF48/50*, *SmTTF62/63*) were tandem repeats and the remaining 108 pairs were segmental repeats. The results indicated that *SmTTF* is highly conserved and that most of the genes may come from the same ancestor. The generation of tandem repeats and segmental repeats was considered to be one of the central mechanisms for the origin of new genes, which also ensured that plants can have a high tolerance to biotic and abiotic stresses (Ober, 2010; Erthmann et al., 2018). The subcellular localization results showed that most of the 78 genes were located in the nucleus, among which *SmTTF05*, *SmTTF39*, *SmTTF40*, *SmTTF55* and *SmTTF78* were located in the chloroplast, and *SmTTF40* and *SmTTF68* were also located in the peroxisome. This implied that *SmTTF* family members may be involved in a variety of biological processes.

We performed a phylogenetic analysis to elucidate the evolutionary relationships between *S. matsudana* and the Trihelix gene family of other species. The 78 Trihelix gene family members of *S. matsudana* were divided into four subfamilies, GT-1, GT-2, SH4 and SIP1, consistent with the results in species such as *Arabidopsis* (Breuer et al., 2009), moso bamboo (Cheng et al., 2019), wheat (Xiao et al., 2019) and Medicago truncatula(Liu et al., 2020a). In contrast to the closely related species poplar, the *S. matsudana* Trihelix genes do not belong to the GTγ subfamily. Studies on the GTγ subfamily genes of rice and *Arabidopsis* revealed that the subfamily genes were mainly involved in the response to stress, with the *Arabidopsis* GTγ subfamily gene *HRA1* responding to submerged dioxygen stress and *OsGTγ1* and *OsGTγ2* both enhancing the tolerance to salt stress in rice (Fang et al., 2010; Giuntoli et al., 2014). In addition, members of the GT-1 and GT-2 subfamilies also exhibited responses to biotic and abiotic stresses. Within the same subfamily, the type and number of motifs of the *S. matsudana* Trihelix proteins are usually similar. In addition, members of the same subfamily usually have similar gene structures and exon numbers. There were differences in gene structure and exon numbers between different subfamilies, with *SmTTF29*, *40*, *64*, *67* and *78* in the GT-1 subfamily containing 5 and 6 exons, respectively. *SmTTF66* in the SH4 subfamily also contained 5 exons, which were significantly different from the exons in other subfamilies. These results suggest that members in the same subfamily may play similar roles in the growth and development of *S. matsudana* and that members with different gene structures or motifs may perform other specific functions.

The function of *SmTTF* may also be influenced by the distribution and type of cis-acting elements on the promoter. In this study, we identified 78 *S. matsudana* Trihelix genes that are involved mainly in light-induced responses and abiotic stresses. In addition to the original study in which the Trihelix gene family was identified for its ability to bind to light response elements on DNA sequences (Nagata et al., 2010), we found that all 78 *S. matsudana SmTTF* promoter regions had at least one light response element present. We found that hypoxic elements were present in the promoter regions of 69 SmTTF genes. In addition, many other cis-acting elements related to drought, low temperature stress and hormone stress were identified, including MBS, LTR, P-box, CGTCA-motif, TGA and GARE. These results suggest that some *S. matsudana* Trihelix genes are involved in the abiotic stress response. These results suggested that evolutionary processes have differentiated the functions of the *S. matsudana* Trihelix gene that play an important role in the response to biotic and abiotic stresses.

Currently, the world is suffering from abiotic stresses such as floods, droughts, and salinization. Stress-responsive genes can be involved in various metabolic processes in plants and help to improve the stress tolerance of plants. Trihelix genes may respond to flooding stress, salt stress, and drought stress (Kaplan-Levy et al., 2012). Flooding stress mainly restricts plant growth by creating a low-oxygen environment and reducing the gas exchange between plants and the outside world. In this study, we found that 84.6% of the *S. matsudana* Trihelix genes had at least one hypoxic response element (ARE) in their promoter regions, which suggests that the *S. matsudana* Trihelix genes may be involved in the response to flooding stress. Therefore, the expression patterns of 78 Trihelix genes at different flooding times in *S. matsudana* were investigated using a self-assayed transcriptome. Many differentially expressed genes were identified, while a large number of Trihelixes exhibited specific expression patterns. In this study, we compared gene expression at five time points and found that expression patterns differed among subfamilies. *SmTTF02*, *06*, *30*, *47*, and *59* in the GT-1 subfamily showed high expression at 4 h after submergence, followed by a decrease in expression, presumably involving the genes of this subfamily in the early submergence stress response. The expression of SH4 subfamily genes was relatively heterogeneous after flooding, and it is assumed that this subfamily is less responsive to flooding stress in *S. matsudana*. A hypoxia-inducible GTγ gene, *HRA1* (At3G10040), was reported in *Arabidopsis*, which negatively regulates the ethylene response factor *RAP2.12* and avoids accelerated carbohydrate depletion due to excessive accumulation of *RAP2.12*. Our previous study found that flooding tolerance hub genes were screened in *S. matsudana* using WGCNA, including *SmTTF30* of the GT-1 subfamily. These results demonstrate that *S. matsudana* Trihelix genes are capable of responding to flooding stress, and some of them may play a regulatory role in the stress process.

In addition, our study also found that the *S. matsudana* Trihelix gene can respond to high salt, drought, and low temperature stresses. Previous studies reported that the GT-2 gene *Gh-A05G2067* in cotton can be induced to be expressed under drought stress and salt stress (Magwanga et al., 2019). The tomato GT-1 gene *ShCIGT* was also induced to be expressed under drought stress and low-temperature stress (Yu et al., 2018). Our analysis revealed changes in the expression level of the Trihelix gene in *S. matsudana* under salt stress, drought stress and low-temperature stress. Among these genes, *SmTTF* expression reached the highest level 4 h after 20% PEG6000 treatment, and then the expression level decreased, indicating that the *S. matsudana* Trihelix gene can participate in the early response to drought stress. The GT-1, GT-2 and GTγ genes were strongly induced in rice, tomato and cotton. In our study, GT-1 genes (19, 30, 40, 47) and GT-2 (01, 14, 28) in *S. matsudana* were more sensitive under abiotic stress, except for the GTγ gene.

In previous studies, *SmTTF30* was selected as one of the *S. matsudana* flooding tolerance hub genes by WGCNA (Chen et al., 2022b). We compared the expression of *SmTTFs* in *S. matsudana*, and the *SmTTF30* gene showed higher gene expression after stress. Evolutionary relationships were higher with the *Arabidopsis* GTγ gene *HRA1* (At3G10040) than other DEGs, so we speculate that *SmTTF30* may play a regulatory role in flooding stress tolerance in *S. matsudana*. Antagonistic interactions between RAP2.12 and HRA1 allow for a flexible response to fluctuating hypoxia and are critical for stress survival. Unlike *HRA1*, *SmTTF30* has the exact opposite effect in *Arabidopsis*, enhancing flooding tolerance, which may be caused by the difference in structure. *SmTTF* was induced to be highly expressed after flooding stress in the overexpression lines *Arabidopsis* and was found to grow better than the wild type after 5 days of flooding. Wild-type *Arabidopsis* was found to generally withstand 3-5 days of flooding stress, and transgenic *Arabidopsis* showed a better phenotype than wild type with a longer time (Xie et al., 2015). In our study, the wild type also withstood only 5 days of submergence stress, while the transgenic lines were stronger than it. Our study demonstrates that overexpression of *S. matsudana SmTTF30* enhances flooding tolerance in *Arabidopsis*, but the regulatory mechanism is not clear. The assay of plant cell activity is a direct and effective way to determine the degree of plant injury and test its resistance to stress, so we used Evans Blue staining to determine the flooding tolerance of *Arabidopsis* thaliana (Liu et al., 2020b). The results of Evans Blue staining showed that the cell activity of overexpressed *Arabidopsis* cells was significantly higher than that of wild type after flooding and that wild-type *Arabidopsis* leaves showed yellowing and hyalinization and curled leaf edges after flooding. In both poplar and *Arabidopsis* studies, the Trihelix gene was found to regulate stomatal development and improve the plant exchange rate for gas and water (Weng et al., 2012; Shibata et al., 2018). Overexpression of the wheat Trihelix gene *TaGT2L1D* in *Arabidopsis* increased the number of stomata in *Arabidopsis* leaves and decreased the drought tolerance of the plants (Zheng et al., 2016). We speculate that *SmTTF30* may regulate leaf stomatal development in *Arabidopsis* by increasing the number of stomata, increasing the rate of gas exchange under flooding stress, and increasing the oxygen content in the body to achieve flooding tolerance. Flooding stress leads to a dramatic accumulation of reactive oxygen species in plant cells, and the excess of reactive oxygen species leads to peroxidation of cell membrane lipids, increased permeability, and disruption of normal cellular functions. Plants can promote the scavenging of reactive oxygen species through antioxidant enzyme systems with non-enzymatic antioxidants (Gong et al., 2022). POD and MDA are among the important indicators. Changes in MDA content and POD activity similarly demonstrated that overexpression of *SmTTF30* improved tolerance under flooding stress in *Arabidopsis*. The trends of changes in MDA content and POD activity in maize *ZmEREB180*-regulated flooding tolerance studies in maize seeds were consistent with our experimental results, with significantly lower MDA content and significantly higher POD activity of the transgenic material than the control after flooding stress (Yu et al., 2019). There was a positive relationship between the expression of flooding-responsive genes and flooding tolerance under flooding stress (Zhou et al., 2020). We hypothesized that overexpression of SmTTF30 could affect the expression levels of hypoxia-responsive genes, enhance cellular redox responses, promote the accumulation of related enzymes, and enhance plant flooding tolerance. In conclusion, *SmTTF30* regulates flooding tolerance in plants, but further studies are needed on the molecular mechanism of regulating flooding tolerance.

## Conclusions

In summary, 78 members of the Trihelix gene family were identified in the *S. matsudana* genome and classified into four subfamilies based on phylogenetic relationships. Genes in the same subfamily usually have similar structures and conserved functional domains. The expression profiles of Trihelix genes were studied under submergence, high salt, drought, and low temperature treatments to determine their response to abiotic stresses. Heterologous transformation of *Arabidopsis* demonstrated that overexpression of *SmTTF30* enhanced plant submergence tolerance. These results provide a basis for resolving the role of the Trihelix gene in plant flooding tolerance and its molecular mechanism.

## Data availability statement

The data that support the findings of this study have been deposited into CNGB Sequence Archive (CNSA) of China National GeneBank DataBase (CNGBdb) with accession number CNP0003817 and CNP0002062.

## Author contributions

JY, YC and JZ contributed to conception and design of the study. JY, ZT, WY, QH, YW and MH performed the experments, analyzed the data, prepared figures and tables. HW, GL, BL, YC, JZ reviewed drafts of the paper, and approved the final draft. All authors contributed to the article and approved the submitted version.

## Supplementary material

Supplementary Table 1 Characteristic of SmTTFs. MW, molecular weight. pI, isoelectric point. Subfamily of the SmTTFs were divided by results of phylogenic analysis (**Figure 1**).

Supplementary Table 2 All primers used in this article.

Supplementary Table 3 All Cis-element of promoter.

Supplemental Figure 1 Expression levels of hypoxia-responsive marker genes in *A. thaliana*. **(A)** *AtADH1* **(B)** *AtPCO2* **(C)** *AtSUS1* **(D)** *AtSUS4*. Data were analyzed by one-way analysis of variance followed by Duncan’s test. Different letters represent statistically significant differences (*p* < 0.05).
