# Supplementary figures and images for: Genome-wide characterization and identification of Trihelix transcription factors and expression profiling in response to abiotic stresses in Chinese Willow (Salix matsudana Koidz)
Source: Front Plant Sci. 2023 Mar 3;14:1125519. doi: 10.3389/fpls.2023.1125519 (PMC10020544; doi:10.3389/fpls.2023.1125519)

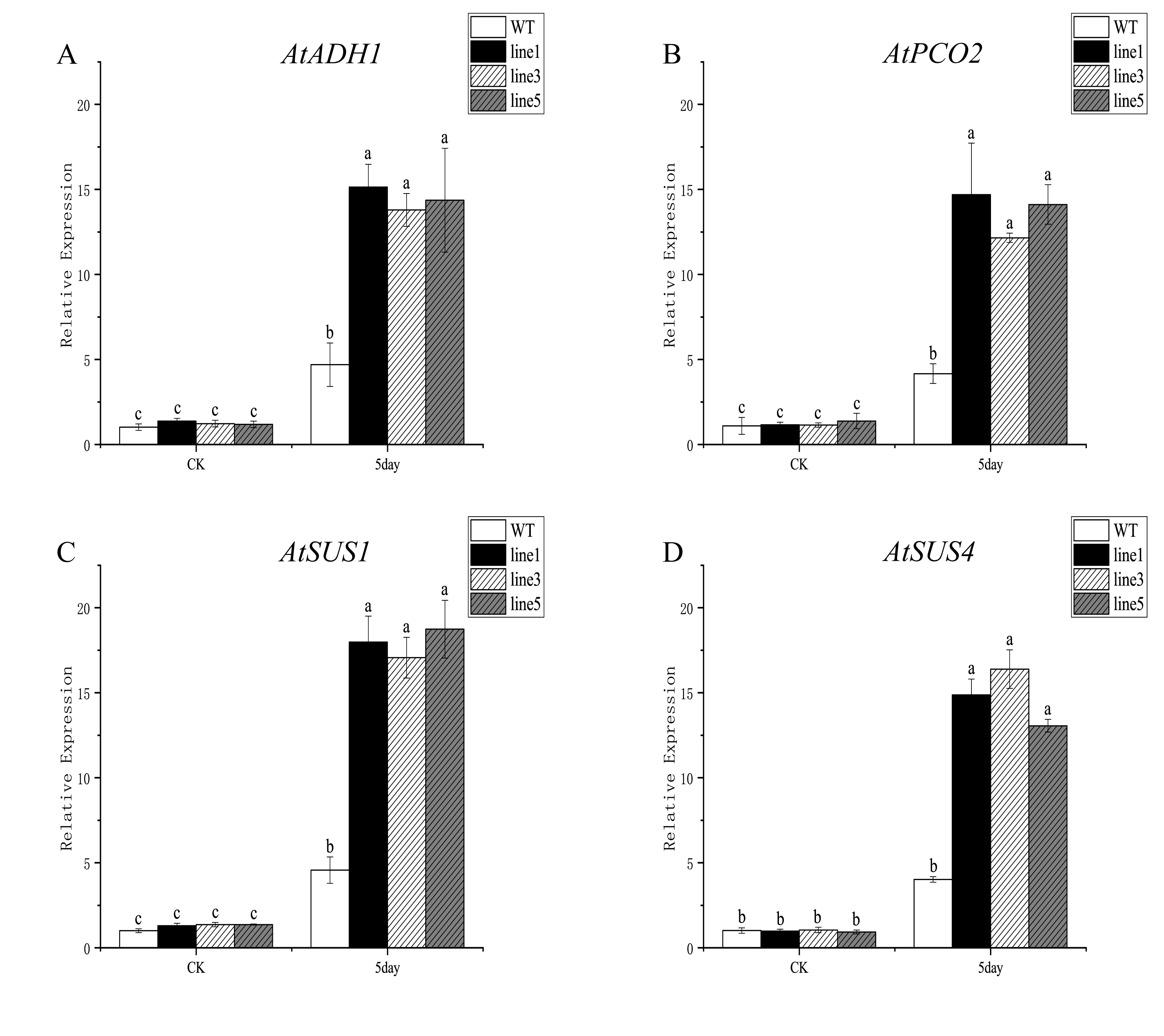

Supplement: Supplemental Figure 1 — Expression levels of hypoxia-responsive marker genes in A. thaliana. (A) AtADH1 (B) AtPCO2 (C) AtSUS1 (D) AtSUS4. Data were analyzed by one-way analysis of variance followed by Duncan’s test. Different letters represent statistically significant differences (p < 0.05). [file Image_1.jpeg]
